# Preparation of Hollow CuO@SiO_2_ Spheres and Its Catalytic Performances for the NO + CO and CO Oxidation

**DOI:** 10.1038/srep09153

**Published:** 2015-03-17

**Authors:** Xiaoyu Niu, Tieying Zhao, Fulong Yuan, Yujun Zhu

**Affiliations:** 1 Key Laboratory of Functional Inorganic Material Chemistry (Heilongjiang University), Ministry of Education, School of Chemistry and Materials, Heilongjiang University, Harbin, 150080 P. R. China

## Abstract

The hollow CuO@SiO_2_ spheres with a mean diameter of 240 nm and a thin shell layer of about 30 nm in thickness was synthesized using an inorganic SiO_2_ shell coating on the surface of Cu@C composite that was prepared by a two-step hydrothermal method. The obtained hollow CuO@SiO_2_ spheres were characterized by ICP-AES, nitrogen adsorption-desorption, SEM, TEM, XRD, H_2_-TPR, CO-TPR, CO-TPD and NO-TPD. The results revealed that the hollow CuO@SiO_2_ spheres consist of CuO uniformly inserted into SiO_2_ layer. The CuO@SiO_2_ sample exhibits particular catalytic activities for CO oxidation and NO + CO reactions compared with CuO supported on SiO_2_ (CuO/SiO_2_). The higher catalytic activity is attributed to the special hollow shell structure that possesses much more highly dispersed CuO nanocluster that can be easy toward the CO and NO adsorption and the oxidation of CO on its surface.

Nowadays, the hollow inorganic functional materials has received much attention compared with solid counterparts because of their low density, high surface area, good surface permeability, large and light-harvesting efficiency^1^,^2^,^3^,^4^,^5^. As a result, hollow structures have been widely applied in catalysis fields such as photocatalysis, hydrogenation, alcohols oxidation, lipases immobilized, methane decomposition, the oxygen reduction reaction et al.^6^,^7^,^8^,^9^,^10^,^11^,^12^. Li and co-workers fabricated the ultra-small Ag nanocrystallite-decorated TiO_2_ hollow spheres by using a two-step hydrothermal method to improve photocatalytic performance^6^. Liu and co-workers developed a novel monolithic catalyst with porous hollow silica nanoparticles for selective hydrogenation reactions^8^. Ravat and co-workers prepared palladium-supported boron-doped hollow carbon spheres as catalysts for the solvent-free aerobic oxidation of alcohols^9^. Tang's group successfully fabricated the hollow zeolitically microcapsulized (HZMC) catalysts with encapsulated noble metal nanoparticles. The HZMC catalysts exhibit good reactant selectivity due to the existence of a zeolitic shell^13^,^14^. Thus, a wide variety of functional materials with SiO_2_ hollow structure can provide more opportunities for catalysis fields due to their intrinsic structural characteristics.

Considering their wide applications, many different methods have been adopted to synthesize hollow spheres containing transition metal^15^,^16^,^17^. Among these preparation methods, the template method is taken for the conventional method to achieve hollow spheres. The templates could be removed by chemical etching or by calcination. Carbonaceous spheres have been usually considered as sacrificial templates to fabricate hollow spheres because of their facile removal^18^,^19^,^20^,^21^. Copper compounds are chosen as primary catalyst component due to their high activity for NOx removal, CO oxidation, alcohol dehydrogenation and vapor-phase hydrogenation^22^,^23^,^24^,^25^,^26^,^27^. In our previous study, the Cu@C composite spheres were prepared by a one-step hydrothermal carbonization (HTC) method and their use as sacrificial templates to synthesize a CuO@SiO_2_ structure^28^. However, the mean diameter of the CuO@SiO_2_ spheres is about 500 nm leading to the possible limitation for its property and application, which is attributed to the large size of the Cu@C composite spheres (about 370 nm) prepared by the one-step HTC method. Moreover, the amount of Cu in CuO@SiO_2_ is higher as 36% using Cu@C as hard template synthesized by the above method. Thus, it is necessary and interesting to explore the controllable synthesis of much smaller CuO@SiO_2_ nanoparticles with low Cu content in order to extend its application.

In the present work, we focused on the synthesis of Cu@C composites by two-step HTC method to obtain the much smaller nanoparticles and lower Cu amount than that of the one-step HTC method. What's more, the hollow CuO@SiO_2_ spheres were prepared by using the Cu@C composite as a hard template. In addition, CO oxidation and NO + CO reactions were used to evaluate the catalytic performance of the prepared hollow CuO@SiO_2_ sample, furthermore, which the effects of hollow shell structure of CuO@SiO_2_ spheres on the catalytic activity were investigated by comparison with CuO supported on silica gel (CuO/SiO_2_) as reference.

## Results

The hollow CuO@SiO_2_ sphere with a layer of CuO in the SiO_2_ interior surface was prepared using a recently developed procedure involving silica encapsulation via using Cu@C composite spheres as hard template^28^. In order to obtain much smaller dimension of the hollow CuO@SiO_2_ sphere, two step hydrothermal carbonization here using glucose and cupric acetate as copper source was brought forward to prepare the smaller Cu@C composite spheres. Different diameter Cu@C composite spheres could been synthesized by controlling the hydrothermal time and the molar ratio of cupric acetate monohydrate (Cu(Ac)_2_·H_2_O) to glucose (the SEM images are presented in Figure S1 and Figure S2 of the Supplementary Information (SI)). The uniform Cu@C composite spheres with a smooth surface and the mean diameter of 210 nm can be prepared under 0.5 h in the first step and 12.0 h in the second step (Figure S3a and Figure S4a in the SI). SEM results demonstrate that the mean diameter of the Cu@C composite spheres is smaller than that of the sample synthesized by one step method (about 370 nm) in our previous study^28^. The reasons for the smaller size of the Cu@C and higher Cu dispersity were analyzed and shown in the SI.

Here, a hollow shell structured CuO@SiO_2_ sample could be obtained by using Cu@C as hard template. A SiO_2_-supported CuO sample (CuO/SiO_2_) was included in the study as reference. Figure S3b and S3c (in the SI) show the XRD diffraction patterns of the CuO@SiO_2_ and CuO/SiO_2_ samples, respectively. The typical diffraction peaks of monoclinic CuO (JCPDS Card No. 05-0661) are observed, which indicates that copper species in the material is CuO. The particle size of the CuO calculated by using Scherrer's formula is 19.8 and 15.4 nm for CuO@SiO_2_ and CuO/SiO_2_ samples, respectively (listed in Table 1).

The morphology of the CuO@SiO_2_ precursor, CuO@SiO_2_ and CuO/SiO_2_ samples were investigated by SEM (Figure S4 of SI). The SEM image of the as-obtained CuO@SiO_2_ precursor shows that Cu@C is coated with silica (Figure S4b). Compared with the Cu@C sample (Figure S4a), the mean diameter of the CuO@SiO_2_ precursor is approximately 240 nm, and the coarse shell consists of unequal nanosized spheres that are generated by the hydrolyzate of TEOS, and the shell thickness is calculated to be about 30 nm. Figure S4c shows the open-mouthed sphere could be observed for the CuO@SiO_2_ sample, which reveals the formation of the hollow sphere structure that is caused by the evolution of CO_2_ during the removal of the internal carbon spheres at 600°C. However, the CuO/SiO_2_ sample is composed of tiny particles, and a few agglomerate spheres also can be seen occasionally (Figure S4d). Thus, the hollow CuO@SiO_2_ spheres with the mean diameter of 240 nm are fabricated by using the Cu@C composite spheres as hard template.

**Figure 1:**
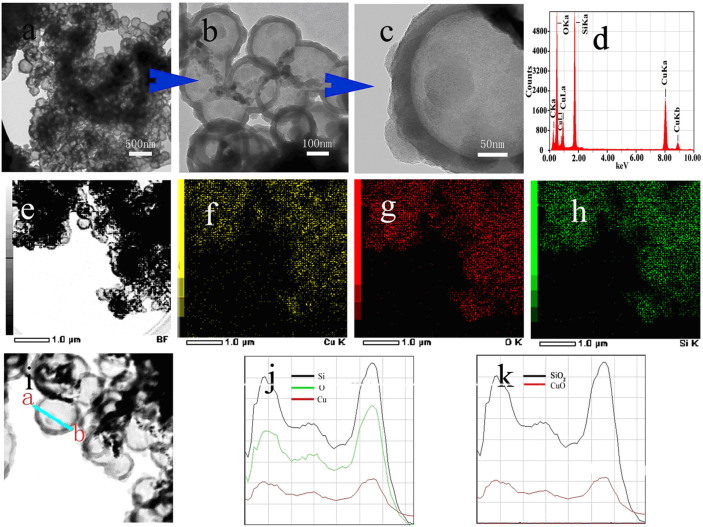
TEM images (a, b, c and e) of CuO@SiO_2_. Also shown are EDX mapping results (d, h) of CuO@SiO_2_ and Line scanning profiles (j, k) of recorded along the line of the CuO@SiO_2_ in image (i).

The TEM image shows that the CuO@SiO_2_ sample exhibits a hollow sphere structure with a mean diameter of 240 nm and the shell of a thin layer of 30 nm in thickness (Figure 1a–c). The EDX spectrum of the CuO@SiO_2_ spheres (Figure 1d) exhibits the presence of strong signals of Cu, O, and Si elements (the scanned region from Figure 1e area), and the Cu content is about 26%, which is in agreement with the ICP results (Table 1). The mapping results of the Figure 1e zone are presented in Figure 1f, g, h, in which the distributions of Cu, Si and O elements are the very same. It indicates that the Cu element is evenly dispersed among Si and O elements, demonstrating the composition of the CuO@SiO_2_ shell structure with Cu, O and Si. In order to further confirm the shell composition, the line scanning of a CuO@SiO_2_ sphere was performed along the line of the CuO@SiO_2_ sphere from a to b shown in Figure 1i. It clearly reveals that the Cu, O and Si elements can be detected in the shell layer (Figure 1j), and the changed trends of the three elements are exactly the same, while any element can not be detected at the sphere center. This further confirms that the unique shell structure of the hollow CuO@SiO_2_ sphere consists of Cu element in the SiO_2_ interior. Moreover, line scanning profiles of CuO and SiO_2_ recorded are the same in Figure 1j to further prove the composition of the CuO@SiO_2_ shell with CuO and SiO_2_ (Figure 1k). However, the lattice fringe of copper oxide phase was not observed by HRTEM, which may be attributed to CuO embedded into the SiO_2_ shell and is not able to be easily observed. Considering the XRD and TEM results, it is confirmed that the CuO uniformly inserted into SiO_2_ layer composes the shell of the hollow CuO@SiO_2_ spheres. In other word, silica encapsulate the highly dispersed CuO paticles in the shell of the hollow CuO@SiO_2_ spheres.

The nitrogen adsorption-desorption isotherms of CuO@SiO_2_ and CuO/SiO_2_ samples are shown in Figure S5 of SI in the Supplementary Information. A typical IV-type isotherms indicates they exhibits mesoporous character^29^. Besides, for the CuO@SiO_2_ and CuO/SiO_2_, the BET surface area is measured to be 85 and 128 m^2^ g^−1^, pore volume is measured to be 0.1 and 0.4 m^3^ g^−1^ and pore size is measured to be 4.9 and 14.9 nm, respectively. Although the surface area of the CuO@SiO_2_ shows much lower than that of the CuO/SiO_2_, but it is larger than what is mentioned in the literature^28^.

**Figure 2:**
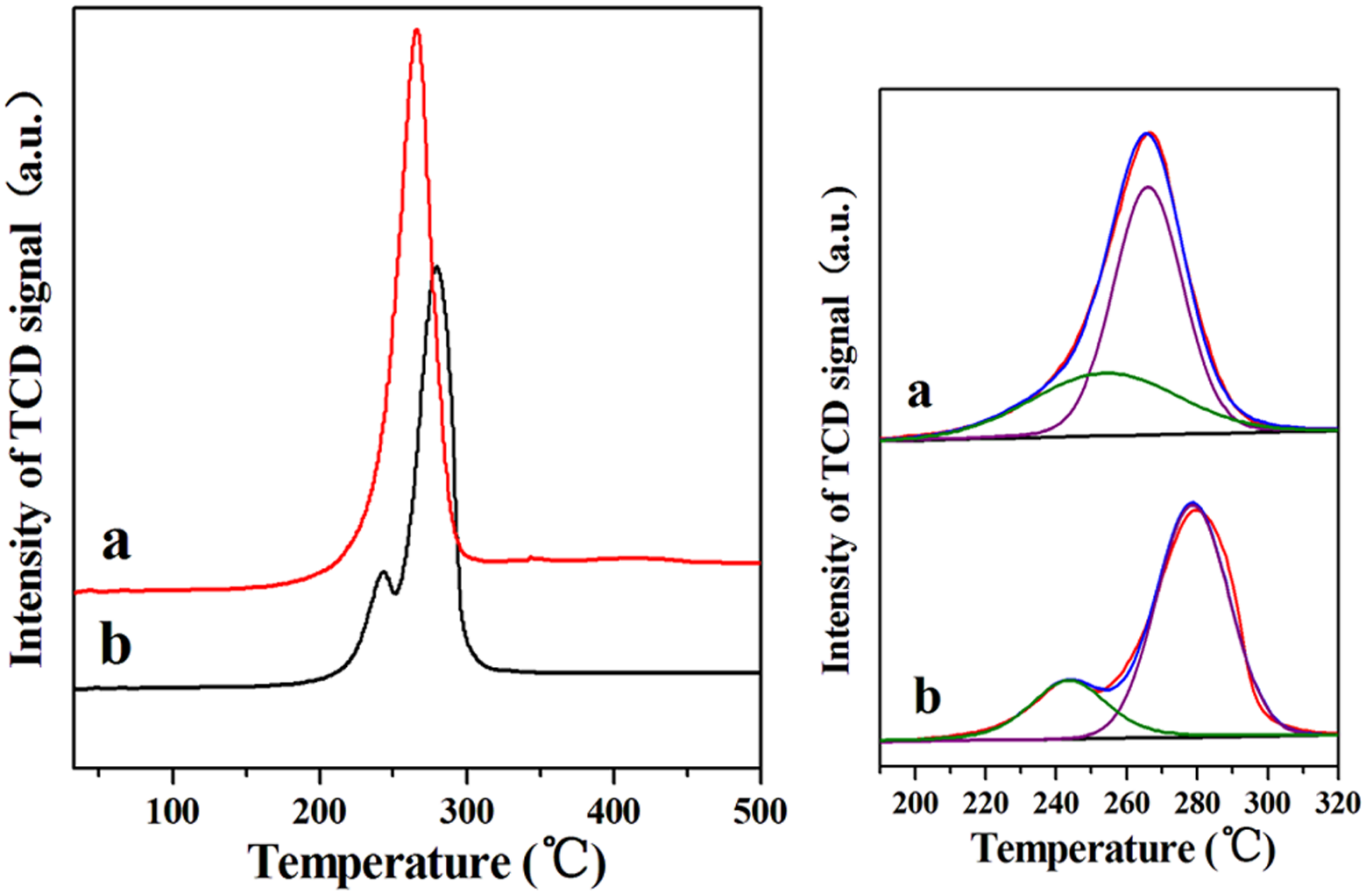
H_2_-TPR profiles of (a) CuO@SiO_2_ and (b) CuO/SiO_2_.

Figure 2 shows the H_2_-TPR profiles of the CuO@SiO_2_ and CuO/SiO_2_. As can be seen, the CuO@SiO_2_ is reduced in a wide range from 160 to 310°C with the peak presented at 266°C, and the H_2_ consumption amount of the referred peak is 4.53 mmol·g^−1^. A shoulder peaks at 254°C can be well fitted in the H_2_-TPR curve for the CuO@SiO_2_. Similar to CuO@SiO_2_, CuO/SiO_2_ also shows a main reduction peak at 278°C and a small shoulder peak at 243°C, and the total H_2_ consumption amount of the referred peak is 3.74 mmol·g^−1^. Besides, the results of H_2_-TPR profiles show that the total H_2_ consumption amount of CuO@SiO_2_ is higher than that of the CuO/SiO_2_. The H_2_ consumption amount is consistent to the CuO amount in the samples. According to the literature^30^,^31^,^32^, the first reduction peak for the two samples can be attributable to the reduction of highly dispersed CuO nanocluster with a smaller size^31^. The second reduction peak at <300°C should be assigned to the reduction of crystalline CuO species which has been detected by XRD though the single peak of the bulk CuO is at about 340°C^33^,^34^. The much lower reduction peak of crystalline CuO species for CuO@SiO_2_ sample can be ascribed to a relativly small CuO size than bulk CuO.

The CO-TPR profiles of the CuO@SiO_2_ and CuO/SiO_2_ are showed in Figure S6 of SI. The wide and obvious CO_2_ peaks could be observed for CuO@SiO_2_ in the range from 150°C to 400°C with the three main peaks presented at 222, 246 and 349°C, which was ascribed to the contribution of the high active surface copper oxide with the different size to react with CO^35^,^36^,^37^. For the CuO/SiO_2_, the shape of CO_2_ peaks is similar to that of the CuO@SiO_2_, which also exhibits three main peaks presented at 309, 390 and 463°C that are formed at higher temperature compared with the CuO@SiO_2_. It is deduced that the CuO@SiO_2_ would be the preferable for CO adsorption and reduced by CO more easily, which is consistent with the H_2_-TPR results.

**Figure 3:**
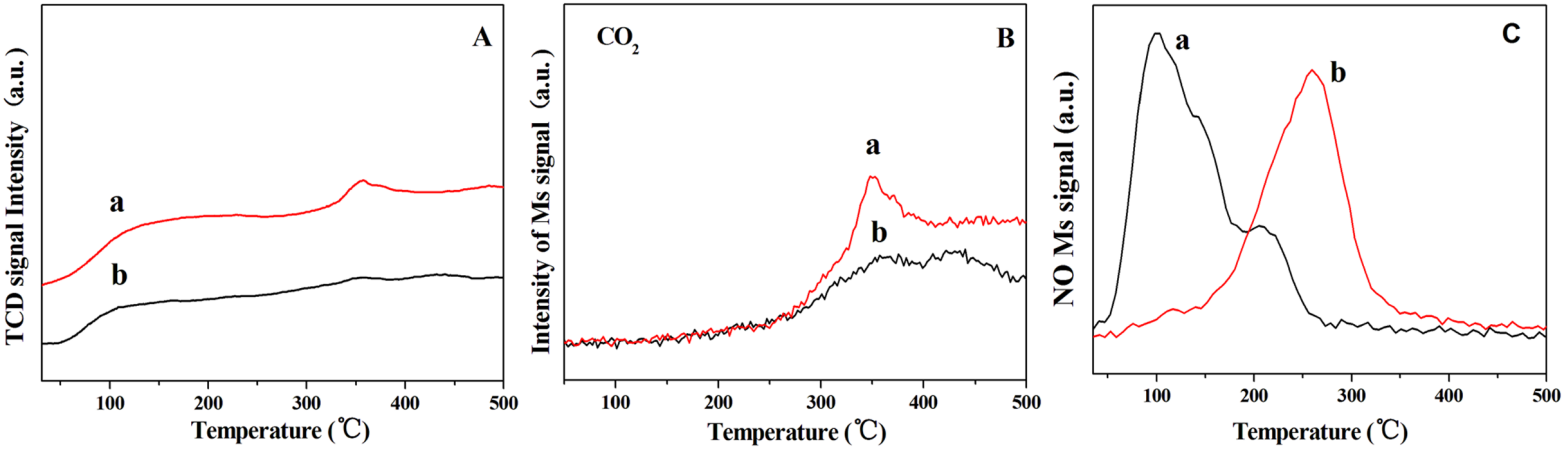
CO-TPD (A, B) and NO-TPD (C) profiles of (a) CuO@SiO_2_ and (b) CuO/SiO_2_.

Figure 3 represents the CO-TPD and NO-TPD profiles of the CuO@SiO_2_ and CuO/SiO_2_. In the CO-TPD, a wide desorption peak is observed in a range of 50–200°C shown in Figure 3A, which is attributed to the CO desorption on the sample surface. Moreover, it is clearly seen that the amount of the CO desorption for the CuO@SiO_2_ is higher than that of the CuO/SiO_2_. At high temperature, another desorption peak at about 360°C is also exhibited for the CuO@SiO_2_ sample. A CO_2_ peak was observed at the same temperature zone (about 360°C) presented in Figure 3B that is formed by the oxidation of the adsorbed CO with the surface oxygen^38^. In contrast to the CuO@SiO_2_, the desorption area of CO_2_ over the CuO/SiO_2_ is much smaller. It reveals that CO can be oxidized on the surface of the CuO@SiO_2_ more easily than that of CuO/SiO_2_. This result is coincident with CO-TPR results in Figure S5, where CuO@SiO_2_ is reduced by CO more easily.

In the NO-TPD profiles, simply, a wide and obvious NO desorption peaks could be observed at 100°C for the CuO@SiO_2_, whereas the NO desorption peak is at 250°C for the CuO/SiO_2_ (Figure 3C). It demonstrates that the NO desorption of the CuO@SiO_2_ is easier than that of the CuO/SiO_2_. Moreover, the area of NO desorption peak is also much larger for the CuO@SiO_2_. Therefore, the lower desorption peak temperature and higher desorption peak area of the hollow CuO@SiO_2_ sample indicate the favorable adsorption for NO.

**Figure 4:**
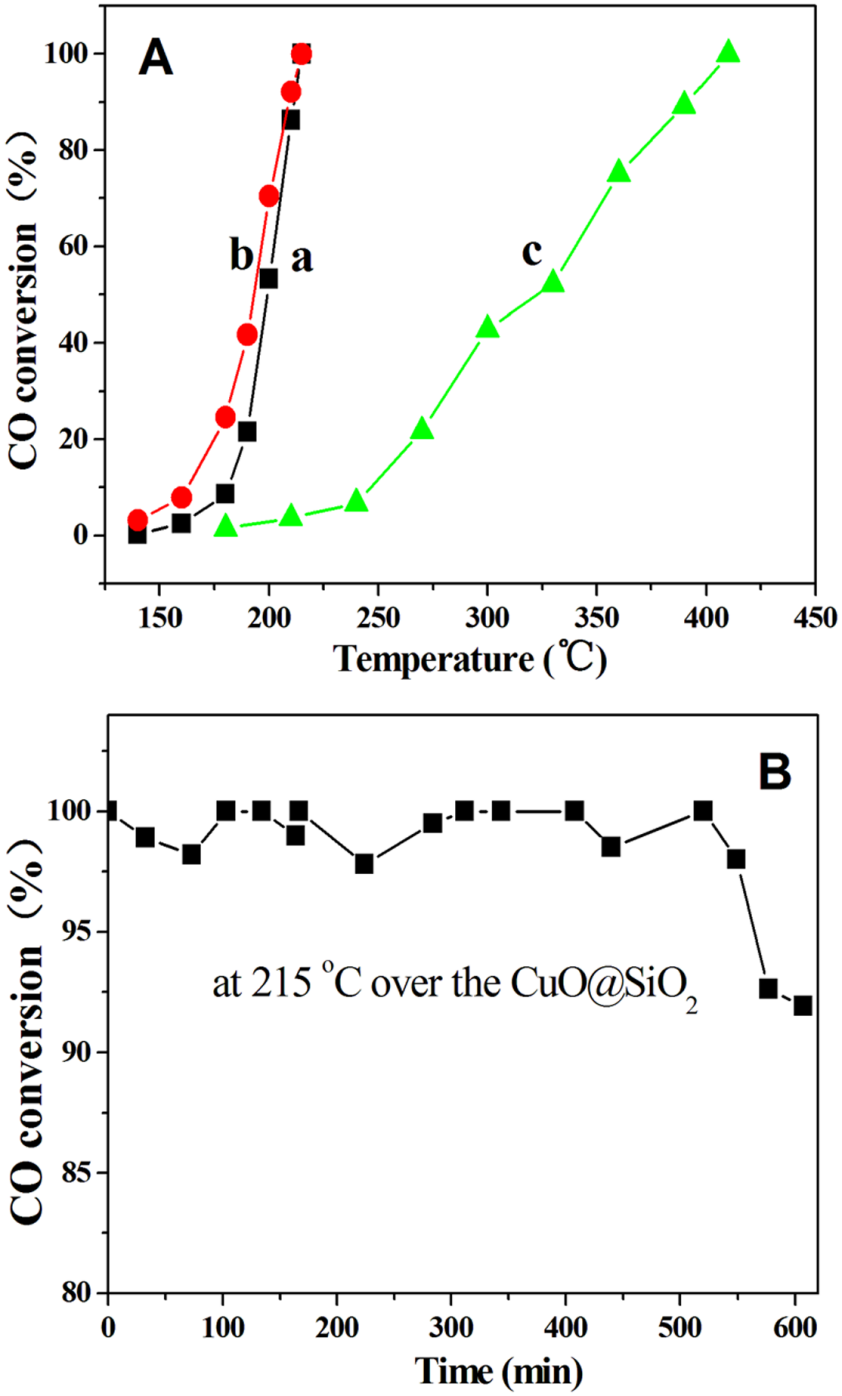
catalytic activities of CO oxidation over the (a) CuO@SiO_2_ (fresh), (b) CuO@SiO_2_ (used) and (c) CuO/SiO_2_.

CuO is one of the most prevalent catalyst compositions in the fields of exhaust emission control and fuel cells^27^, so the catalytic performance of the hollow CuO@SiO_2_ sample has been evaluated by using CO oxidization and NO + CO reaction. CuO/SiO_2_ exhibits poor activity in CO oxidation, while the activity of the hollow CuO@SiO_2_ spheres is evidently enhanced, confirming the essential role of the hollow shell structure in CO oxidation. As shown in Figure 4A(a), (c), it is found that the conversion of CO increases with reaction temperature for both the CuO@SiO_2_ and CuO/SiO_2_. For the CuO@SiO_2_, CO can be completely converted to CO_2_ at 215°C, whereas CO is at the first stage of conversion, the conversion is only about 4% at 215°C, and the complete conversion of CO is achieved at 410°C over the CuO/SiO_2_. A reused test has also been performed to study the stability of the CuO@SiO_2_ sample by repeated evaluating the CO oxidation activity from room temperature to high temperature over the used CuO@SiO_2_. It is found that the used CuO@SiO_2_ shows reasonably stable activity for CO oxidation (Figure 4A(b)). After the second successful test, the used CuO@SiO_2_ still maintains a near 100% conversion of CO to CO_2_ at 215°C. The catalytic performance of the hollow CuO@SiO_2_ sphere was also carried out under long-term high-temperature catalytic conditions. The result shows that the activity of CuO@SiO_2_ catalyst can maintain a 100% conversion of CO, and no deactivation trend occurs during 540 min at 215°C (Figure 4B). For the CuO@SiO_2_ sample prepared from Cu@C by using one step HTC method in the CO oxidation, it exhibits a little higher catalytic activity than the hollow CuO@SiO_2_ sphere prepared in this study (Cu@C obtained by two step hydrothermal), which is a near 100% conversion of CO to CO_2_ at 210°C (Figure S7a of SI) due to the high Cu amount (36% Cu). The hollow zeolitically microcapsulized (HZMC) catalyst with encapsulated CuO nanoparticles (CuO@S1) as reference was also prepared^13^,^14^. But, the Cu amount of the CuO@S1 sample was determined to a maximum of 12.7% by ICP less than 26% of the hollow CuO@SiO_2_ sphere sample. The activity of CO oxidation for CuO@S1 is a near 100% conversion of CO to CO_2_ at 300°C, which shows much lower than that of the hollow CuO@SiO_2_ sphere sample (Figure S7b of SI). All results indicate the hollow CuO@SiO_2_ sphere sample exhibits much higher catalytic activity and stability for the CO oxidation reaction.

**Figure 5:**
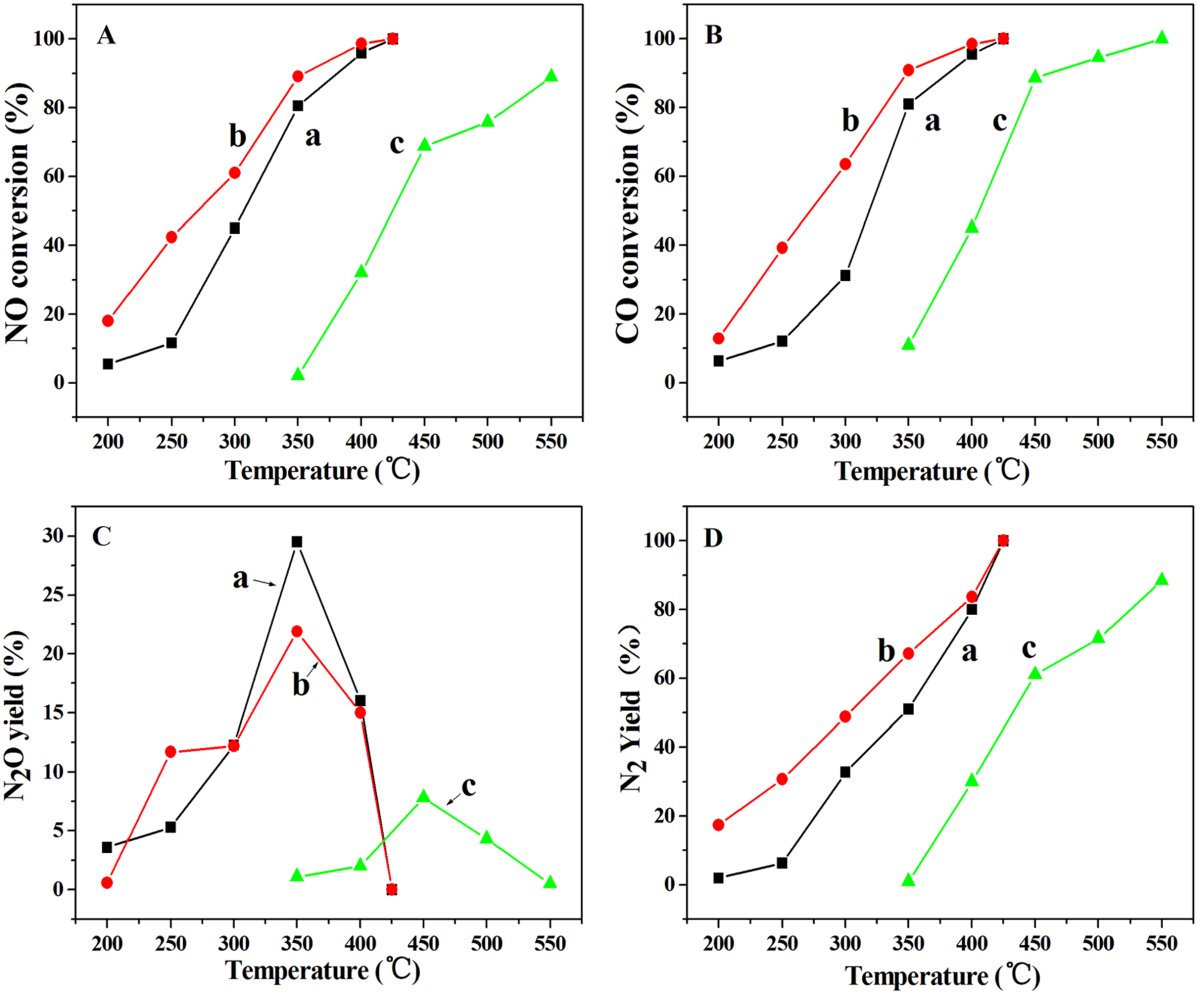
catalytic activities of NO + CO reaction over the (a) CuO@SiO_2_ (fresh), (b) CuO@SiO_2_ (used) and (c) CuO/SiO_2_.

Figure 5 shows the catalytic activities of the CuO@SiO_2_ and CuO/SiO_2_ samples as a function of temperature for NO + CO reaction. The catalytic activity increases with the reaction temperature from 200°C to 425°C and dramatically increases just for above 250°C over the CuO@SiO_2_ (Figure 5A, B). N_2_O is mediates for for both the CuO@SiO_2_ and CuO/SiO_2_ (Figure 5C). However, the CuO/SiO_2_ exhibits very low activity, especially at the reaction temperature of below 350°C, almost no conversion of NO is observed (Figure 5A). When the temperature increases to 425°C, the activity and selectivity change so much that NO is converted completely to N_2_ for the CuO@SiO_2_, while the conversion of NO and yield of N_2_ over CuO/SiO_2_ sample are only about 60% and 46%, respectively (Figure 5D). Furthermore, an activity test of the used CuO@SiO_2_ has been performed to evaluate the catalytic stability. It is obvious found that the reused CuO@SiO_2_ also shows very stable activity. For the used CuO@SiO_2_, the conversion of NO and the yield of N_2_ at 250°C are 42.4% and 30.7%, respectively. Notably, above 250°C, the conversion of NO and CO increases dramatically and reaches 100% at 425°C. The activity of the used CuO@SiO_2_ does not decrease without any treatment after first test (Figure 5A). In contrary, the activity of the used CuO@SiO_2_ is higher than that of the fresh CuO@SiO_2_, which may be attributed to the activation for the CuO@SiO_2_ in the first test. However, the temperature of the complete conversion for NO and CO does not change over the used CuO@SiO_2_. Compared with the CuO/SiO_2_ catalyst, the CuO@S1 catalyst displays much higher catalytic activity for NO + CO though it contains low Cu amount (Figure S8). The conversion of NO and CO reaches near 100% at 500°C. However, the CuO@S1 catalyst still shows much lower NO + CO activity than the hollow CuO@SiO_2_ sphere sample. Therefore, it further confirms that the hollow CuO@SiO_2_ structure exhibits much higher activity and stability in NO + CO reaction.

## Discussion

According to the results of XRD, TEM, SEM and nitrogen adsorption-desorption, the hollow CuO@SiO_2_ spheres with a mean diameter of 240 nm and the shell layer of 30 nm are successfully synthesized. The hollow CuO@SiO_2_ spheres exhibit much higher activity and stability than CuO/SiO_2_ for the CO oxidation (Figure 4) and CO + NO reactions (Figure 5). Even though CuO@SiO_2_ showed lower activity for CO oxidation compared with the Co_3_O_4_^39^ and CuO-CeO_2_^40^, the catalytic activity of the CuO@SiO_2_ is better than that of the CuO supported on SiO_2_ hollow spheres^41^ and SBA-15^42^. Song et. al. also reported that CuO supported on SiO_2_ hollow spheres showed good activity for CO oxidation compared with the CuO supported on commercial SiO_2_, which complete oxidation of CO was at 250°C over 20% CuO/SiO_2_ hollow spheres. However, CuO supported on SBA-15 exhibited low catalytic activity of CO oxidation. Thus, it suggests that the support structure has a great influence on the catalytic activity for CO oxidation. However, CuO@SiO_2_ synthesized in this paper exhibited much lower activity for NO + CO reaction than that CuO@SiO_2_ prepared from Cu@C composites using one-step HTC method in our previous study^28^. It may be attributed to the difference in the Cu amount in the two samples, which the Cu amount in CuO@SiO_2_ is 26% and 36% for this paper and previous paper, respectively. Moreover, the flow rates of both NO and CO are 25 mL/min instead of 23 mL/min in the NO + CO reaction^28^. The CuO@SiO_2_ exhibits a noticeable well activity, which is possibly originated from the formation of specific active sites for its hollow sphere structure. From the H_2_-TPR results, it is clearly seen that the CuO@SiO_2_ can be reduced at a much lower temperature than that of CuO/SiO_2_. Moreover, the first reduction peak area is 3640 and 2046 for the CuO@SiO_2_ and CuO/SiO_2_ (Table 1), respectively, which indicates the presence of much more highly dispersed CuO nanocluster in the hollow CuO@SiO_2_ that may be conducive to the catalytic activity. The CO-TPR results also demonstrate that the hollow CuO@SiO_2_ possesses much more highly dispersed CuO that would be preferable for CO adsorption and reduced by CO more easily. In contrast to the CuO@SiO_2_, the desorption area of CO_2_ over the CuO/SiO_2_ is much smaller (Figure 3B). It reveals that CO can be oxidized on the surface of the CuO@SiO_2_ more easily than that of the CuO/SiO_2_. This result is coincident with CO-TPR results in Figure 2, where CuO@SiO_2_ is reduced by CO more easily. In addition, the CO-TPD results reveal that CO can more easily adsorb and be oxidized on the surface of the CuO@SiO_2_, which indicates high catalytic activity of CO oxidation for the CuO@SiO_2_ sample combined with the H_2_-TPR and CO-TPR results. Besides, the NO-TPD results indicate that the lower desorption peak temperature and the higher desorption peak area of the hollow CuO@SiO_2_ are beneficial to catalytic activity of CO + NO reaction. Therefore, the specific hollow structure of the CuO@SiO_2_ possesses much more highly dispersed CuO nanocluster that leads to be more easy adsorption and reaction for CO and NO, which results to the high catalytic activity.

## Conclusions

A two-step HTC method was used to prepare the Cu@C composite spheres that showed much smaller size than using one-step HTC method. Thus, the much smaller hollow CuO@SiO_2_ spheres were successfully synthesized using a hard template of the Cu@C composites prepared by two-step HTC method. The mean diameter and thin shell layer in thickness of the hollow CuO@SiO_2_ spheres are about 240 nm and around 30 nm, respectively. And the amount of Cu decreased from 36% to 26% compared with CuO@SiO_2_ sample in our previous study. The hollow CuO@SiO_2_ spheres exhibited particular catalytic activities of CO oxidation and NO + CO reactions compared with CuO supported on the commercial silica gel (CuO/SiO_2_). The higher catalytic activity is attributed to the hollow structure that possesses much more highly dispersed CuO nanocluster that can be more easily for CO and NO adsorption and oxidation of CO on its surface.

## Methods

### Preparation of copper@C composites

The preparation of the Cu@C composites spheres was put forward by using the two-step HTC method. A detailed synthesis procedure is as follows: 1.89 mmol D-Glucose monohydrate (C_6_H_12_O_6_·H_2_O) was dissolved in 30 ml distilled water to form a clear solution, then the clear solution was transferred into a 50 mL capacity Teflon-lined stainless steel autoclave, which was then sealed and heated at 180°C for 4 h. The autoclave was cooled to the room temperature, and then 1.89 mmol cupric acetate monohydrate (Cu(Ac)_2_·H_2_O) was added and stirred for 0.5 h. The mixture was transferred into the autoclave again, which was then sealed and heated at 180°C for 12 h. The solid products were separated by centrifugation, washed with the distilled water and absolute ethanol for several times, and finally dried at 80°C for 8 h. The as-prepared samples are denoted as Cu@C.

### Preparation of hollow CuO@SiO_2_ sphere

Typically, 0.40 g prepared Cu@C was dispersed in a solution that is composed of 75 mL ethanol, 10 mL H_2_O, 3.8 mL NH_3_·H_2_O (25 wt%) and 0.264 g n-Hexadecyl-triammonium bromide (C_16_H_33_(CH_3_)_3_NBr). After being stirred at room temperature for 0.5 h, the solution was dispersed by ultrasound for 0.5 h, and then 0.4 mL tetraethyl orthosilicate (TEOS) was added dropwise to the reaction mixture by using a 1 mL syringe. Then, the mixture was stirred at room temperature for 8 h, and the stir speed was 500 rpm. The solid product was separated by centrifugation, washed with distilled water and absolute ethanol for three times, and the sample precursor was obtained after being dried at 80°C for 6 h. Finally, the hollow structure was gotten after being calcined under atmosphere at 600°C for 6 h. The as-prepared samples are denoted as CuO@SiO_2_.

### Preparation of CuO/SiO_2_ sample

CuO/SiO_2_ sample was prepared starting with a suspension of 1.0 g dried silica gel (hydrophilic-200 Silicon dioxide (99.8%), Aladdin, 200 m^2^/g, <100 mesh) in 50 mL distilled water and a solution of cupric acetate (Cu(Ac)_2_) (10 mL 0.5 mol/L). After being stirred at room temperature for 3 h, Cu(Ac)_2_ solution was exclusively precipitated by adding an aqueous solution of NaOH (0.05 mol/L) till pH was 6.5. And then the mixture was continuing stirred for 3 h. After filtrating, the obtained solid catalyst precursor was washed with distilled water until pH was 7 and dried at 80°C for 6 h. Finally, the CuO/SiO_2_ sample was obtained after calcining under atmosphere at 600°C for 6 h.

### Characterizations

X-ray diffraction (XRD) patterns were obtained with a D/MAX-3B X-ray Diffractometer (Rigaku Co.), using Cu Kα radiation combined with Ni-filter. The mean diameter of CuO was calculated by using the full width at half maximum of the CuO(111) reflection at 2θ 38.7° and Scherrer's equation (*D* = [*Kλ*]/[*β*cos*θ*], *D* is the mean size of the ordered crystalline domains, *K* is a dimensionless shape factor that has a typical value of about 0.9, *λ* is the X-ray wavelength, *β* is the line broadening at half the maximum intensity, *θ* is the Bragg angle.). The morphology was observed with scanning electron microscopy (SEM) on a Hitachi S-4800 field emission electron microscope operating at 20 kV. Transmission electron microscopy (TEM) measurements were taken on a JEM-2100 electron microscope operating at 200 kV. Nitrogen adsorption-desorption measurements were performed at −196°C using a Micromeritics Tristar 3020 physisorption instrument. The samples were outgassed under vacuum at 150°C for 8 h before the measurement. The pore size distribution was obtained from the analysis of the adsorption branch of the isotherms using the BJH method. Carbon monoxide temperature programmed desorption (CO-TPD) and NO temperature programmed desorption (NO-TPD) were carried out in a full automatic instrument (XQ TP-5080, China) and performed in the following procedure: Firstly, 100 mg of the catalyst was mounted in a quartz tube and calcined under a helium stream (30 mL/min) at 300°C for 1 h. After the catalyst was cooled down to 25°C, 1% CO or 1% NO was introduced into the system at the rate of 20 mL/min for 1 h. Then the catalyst was flushed in He flow (30 mL/min) to remove physisorbed CO or NO at 25°C. Finally, the sample was gradually heated from 25°C to 600°C at a ramp of 10°C/min. The CO/NO desorption was monitored by a thermal conductivity detector (TCD) and MS. H_2_ temperature programmed reduction (H_2_-TPR) and CO temperature programmed reduction (CO-TPR) were performed on full automatic instrument (XQ TP-5080, China) and performed in the following procedure: The samples (30 mg) were treated in an N_2_ stream at 100°C for 1 h. After being cooled to room temperature in the same atmosphere, the samples were swept with 5% H_2_/N_2_ or 5% CO/He (30 mL/min) until the baseline on the recorder remained unchanged. The samples were finally heated in 5% H_2_/N_2_ or 5% CO/He from room temperature to 600°C at a rate of 10°C/min. The H_2_-TPR profiles of the CuO@SiO_2_ and CuO/SiO_2_ samples were fitted using XPSpeak program. The copper content of the prepared samples was measured by inductively coupled plasma atomic emission spectrometry (ICP-AES) on an Optima 7000DV Perkin-Elmer instrument.

### Catalytic activity evaluation

CO oxidation was carried out with a fixed-bed reactor with a 6 mm-diameter glass tube, 0.100 g of the catalyst (40–60 mesh) was set in the reactor by using quartz wool, gaseous mixtures of CO/air = 1/99 were fed to the catalyst bed after being blended at a rate of 20 mL/min. The Weight Hour Space Velocity (WHSV) value is 12000 mL·h^−1^·g^−1^. In any particular run, the data were recorded after at least 1 h on stream being allowed after each temperature change to ensure that the equilibrium of catalytic reaction was reached. The catalyst was cooled down to room temperature after complete conversion of CO over the fresh catalyst. And then, the activity tests were carried out from room temperature to high temperature over the used catalyst without any further treatment. The gas composition was analyzed before and after the reaction by an online gas chromatography using TDX-01 column (2 m × 3 mm). The activity of CO oxidation reaction was evaluated by the following equation:
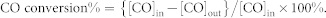


CO + NO reaction was performed with a fixed-bed reactor with a 6 mm-diameter glass tube, 0.100 g of the catalyst (40–60 mesh) was set in the reactor by using quartz wool, gaseous mixtures of 0.8% NO and 0.8% CO diluted with He were fed to the catalyst bed after being blended and the flow rates of NO and CO were both 25 mL/min. The WHSV value is 30000 mL·h^−1^·g^−1^. At different temperature, the data were recorded after at least 1 h on stream being allowed after each temperature change to ensure that the equilibrium of catalytic reaction was reached. The catalyst was cooled down to room temperature after complete conversion of CO and NO over the fresh catalyst. And then, the activity tests were carried out from room temperature to high temperature over the used catalyst without any further treatment. The gas composition was analyzed before and after the reaction by an online gas chromatography, using molecular sieve 5A column for separating NO, N_2_ and CO, and Porapak Q column for separating CO_2_ and N_2_O. The activity of CO + NO reaction was evaluated by the following equations:
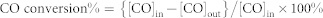

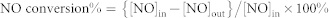

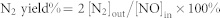

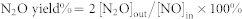
where [CO]_in_ and [NO]_in_ were the concentration of CO and NO measured before the reaction, respectively, whereas [CO]_out_, [NO]_out_, [N_2_]_out_ and [N_2_O]_out_ were the concentration of CO, NO, N_2_ and N_2_O measured after the reaction, respectively.

## Author Contributions

X.N. and T.Z. performed the experimental works, analyzed results. F.Y. assisted in the analyses of results. Y.Z. proposed, planned, and designed the project and reviewed the manuscript prior to submission. All authors wrote the manuscript.

## Supplementary Material

Supplementary Information Supplementary Info

## Figures and Tables

**Table 1:** the texture properties of CuO@SiO_2_ and CuO/SiO_2_

samples	Cu (wt%)	CuO Particle size(nm)	H_2_-TPR Peak temperature (°C)	H_2_-TPR Peak area	H_2_ Consumption (mmol·g^−1^)	Surface area (m^2^·g^−1^)	Pore Volume (m^3^·g^−1^)	Pore Size (nm)
CuO@SiO_2_	26	19.8	254	3640	4.53	85	0.1	4.9
			266	7140				
CuO/SiO_2_	21	15.4	243	2066	3.74	128	0.4	14.9
			278	6595				
