# Supplementary material for: Preparation of Hollow CuO@SiO2 Spheres and Its Catalytic Performances for the NO + CO and CO Oxidation
Source: Sci Rep. 2015 Mar 17;5:9153. doi: 10.1038/srep09153 (PMC4361854; doi:10.1038/srep09153)
Supplement: Supplementary Information — Supplementary Info [file srep09153-s1.pdf]

Supplementary Information

Preparation of Hollow CuO@SiO_2 Spheres and Its Catalytic Performances for the NO+CO and CO Oxidation

Xiaoyu Niu, Tieying Zhao, Fulong Yuan*, Yujun Zhu*

Key Laboratory of Functional Inorganic Material Chemistry (Heilongjiang University), Ministry of Education, School of Chemistry and Materials, Heilongjiang University, Harbin, 150080 P. R. China

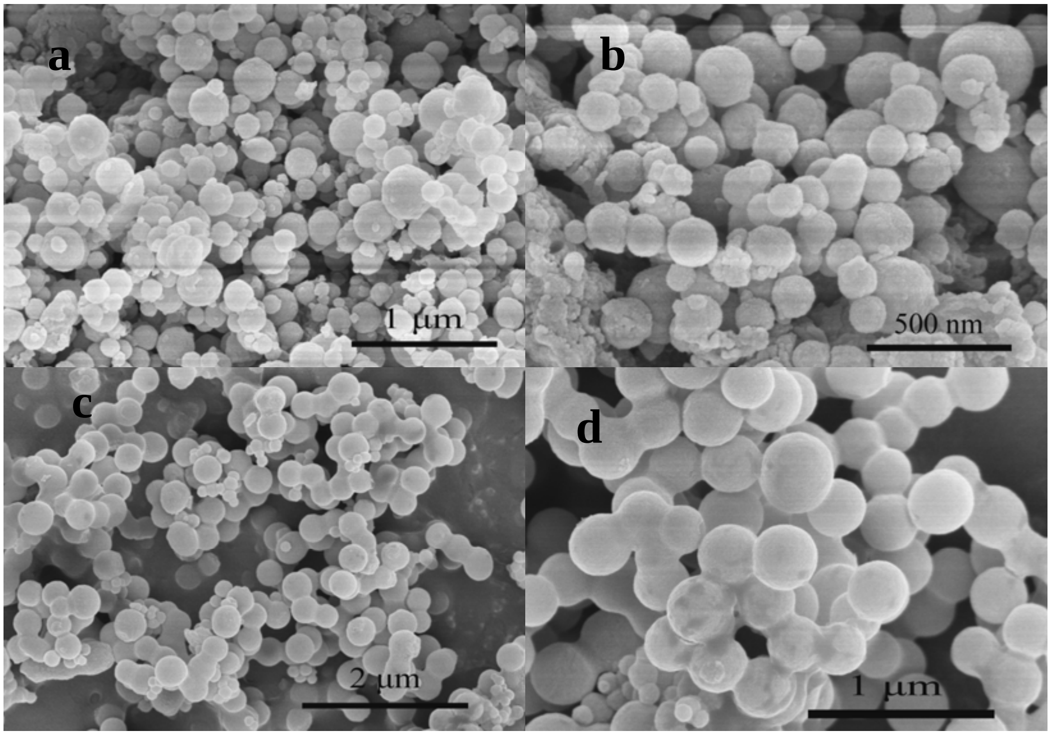

Figure S1. SEM images of Cu@C samples prepared at different hydrothermal time in the second step (a, b: 3.0 h; c, d: 6.0 h)

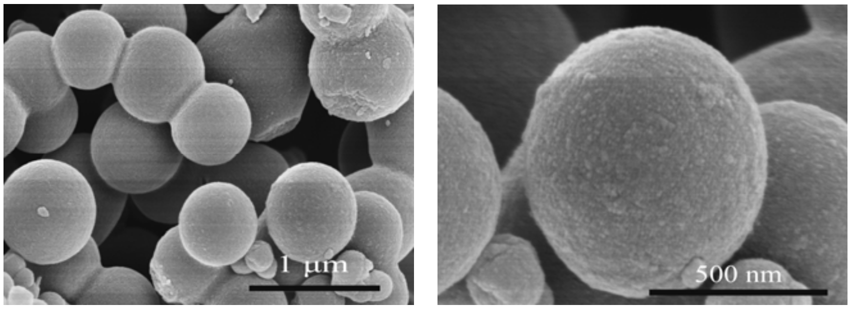

Figure S2. SEM images of Cu@C sample (the molar ratio of cupric acetate monohydrate ($Cu(Ac)_2 \cdot H_2O$) to glucose is 1:5, the hydrothermal time in the second step is 12.0 h).

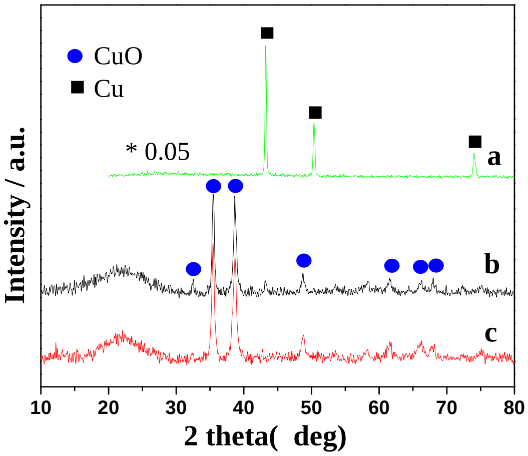

Figure S3.XRD patterns of (a) Cu@C, (b) $CuO@SiO_2$ and (c) CuO/SiO_2

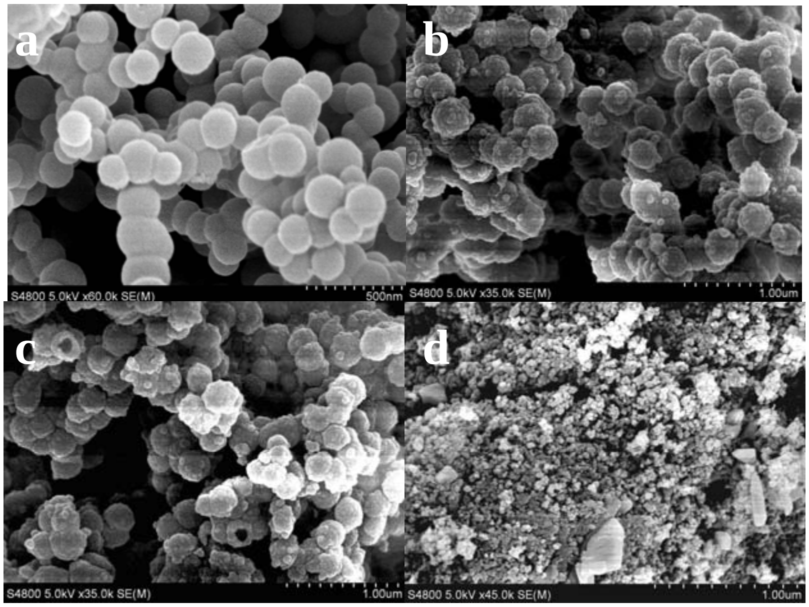

Figure S4. SEM images of (a) Cu@C, (b) $CuO@SiO_2$ precursor, (c) $CuO@SiO_2$ and (d) CuO/SiO_2

The reasons for the smaller size of the Cu@C and higher Cu dispersity using two step method

It is known that in the HTC process, the dehydration and fragmentation of glucose can give rise to different soluble products, such as furfural-like compounds (5-hydroxymethylfurfural, furfural and 5-methylfurfural), organic acids, aldehydes and phenols. Taking into account that the reduction capacity between glucose and its dehydration and fragmentation products might be different, we tentatively treated an aqueous solution of glucose in the first step, and then cupric acetate was added to the resulting solution and continually treated in the second step for a certain period of time in order to control the Cu amount and the size of Cu@C composite. Therefore, base on the differences in reducing capacity and formation rate of the Cu@C composites between the solutions of derivative products generated from glucose and glucose, Cu^{2+} can be reduced slowly to Cu after the glucose was first treated for a period time under HTC process. On the contrary, Cu^{2+} can be reduced much faster in one step method than that of two step method, which leads to that it is difficult to obtain the low Cu amount and well size dispersity by controlling temperature, time and reactant ratio etc. using one step method. Thus, Cu@C composite spheres with a smaller spherical size and much higher Cu dispersity could be obtained via the two-step method. Consequently, the two-step method may open a new route for the synthesis of smaller sized metal carbonaceous materials.

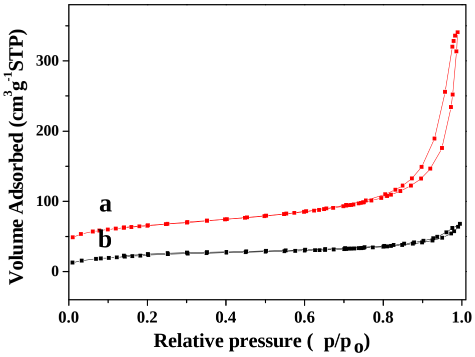

Figure S5. Nitrogen adsorption-desorption isotherms of (a) $CuO@SiO_2$ and (b) CuO/SiO_2

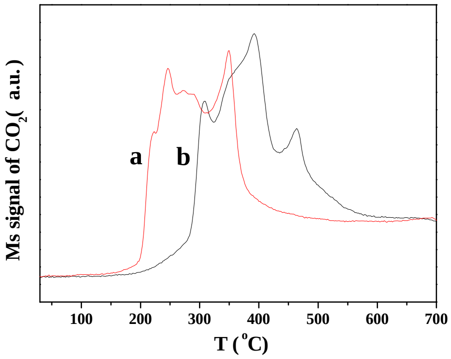

Figure S6. CO-TPR profiles of (a) $CuO@SiO_2$ and (b) CuO/SiO_2

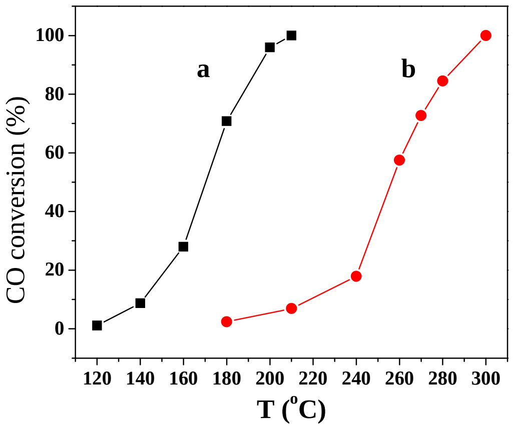

Figure S7 CO oxidation over (a) the $CuO@SiO_2$ prepared from Cu@C by using one step HTC method and (b) CuO@S1

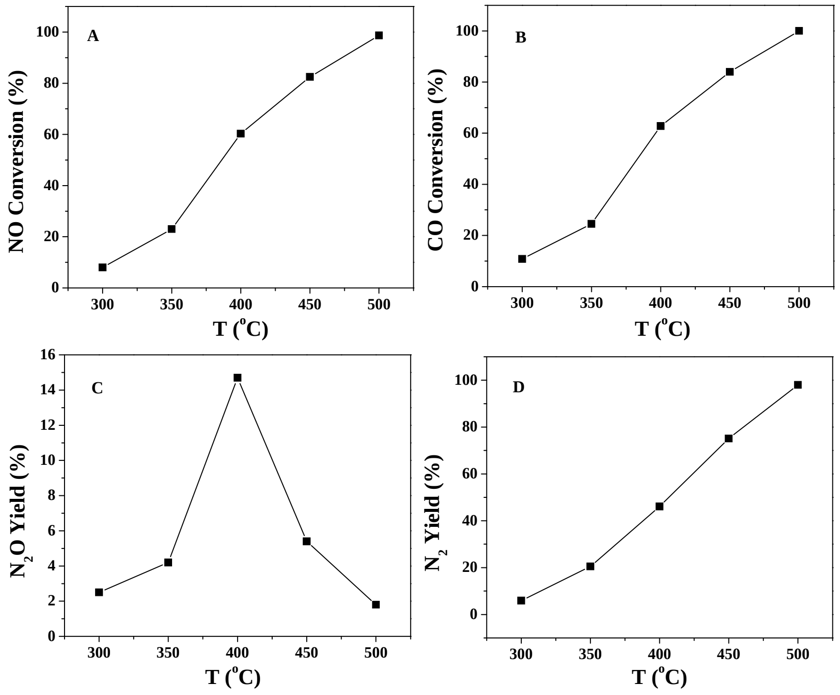

Figure S8 catalytic activities of NO + CO reaction over the CuO@S1
